# Trends and Characteristics of Brown Rice Consumption among Adults in Japan: An Analysis of the National Health and Nutrition Surveys, 2012–2019

**DOI:** 10.3390/nu16101473

**Published:** 2024-05-13

**Authors:** Nayu Ikeda, Miwa Yamaguchi, Nobuo Nishi

**Affiliations:** 1 International Center for Nutrition and Information, National Institute of Health and Nutrition, National Institutes of Biomedical Innovation, Health and Nutrition, 3-17 Senriokashimmachi, Settsu, Osaka 566-0002, Japan; myamaguchi@nibiohn.go.jp (M.Y.); nishi.nobuo.24@slcn.ac.jp (N.N.); 2 Graduate School of Public Health, St Luke’s International University, 3-6-2 Tsukiji, Chuo-ku, Tokyo 104-0045, Japan

**Keywords:** brown rice consumption, National Health and Nutrition Survey, adults, Japan

## Abstract

Brown rice is a familiar whole grain in Japan. We examined national trends in brown rice consumption among Japanese adults aged ≥20 years old, using individual-level data from the National Health and Nutrition Surveys conducted between 2012 and 2019. We employed multivariable logistic regression to identify factors associated with brown rice consumption. The 95th percentile of daily brown rice intake remained at 0.0 g throughout the study period. The percentage of brown rice consumers increased from 1.8% (95% confidence interval: 1.6–2.1) in 2012 to 2.6% (95% confidence interval: 2.0–3.4) in 2019. Compared with individuals who consumed only white rice, brown rice consumers had significantly higher mean intake levels of macronutrients, legumes, vegetables, fruits, and nuts. Brown rice consumption was positively associated with certain sociodemographic characteristics (being female, older age, residing in a major city, living without very young children, and having higher education levels) and health behaviors (lower body mass index, engaging in regular exercise, and being a former or never smoker). Despite its potential nutritional benefits in balanced diets, only a small fraction of adults in Japan consume brown rice, indicating a need for further promotion, particularly among individuals with characteristics associated with brown rice consumption.

## 1. Introduction

Whole grains are rich in dietary fiber, vitamins, and minerals, offering protective benefits against non-communicable diseases. Diets that are abundant in whole grains are globally endorsed as a component of Sustainable Healthy Diets [1] and Planetary Health Diets [2], both of which advocate for sustainable and healthful eating practices for the global population. Previous research has indicated an inverse association between whole grain consumption and the risk of noncommunicable diseases [3,4,5,6], highlighting low intake of whole grains as one of the major dietary risk factors contributing to preventable deaths and disabilities worldwide [7].

In 2010, the global average daily intake of whole grains was approximately 38 g, and Japan was reported to have one of the lowest national daily intake figures, at approximately 8 g [8]. As Japan’s population continues to age, promoting whole grain consumption has emerged as an effective nutrition policy for extending healthy life expectancy and mitigating the increased healthcare costs associated with noncommunicable diseases. Despite the growing recognition of the importance of whole grain intake, it has yet to be officially recommended by any dietary guidelines in Japan [9,10]. This absence of an official recommendation may be partially attributable to the current lack of sufficient evidence regarding Japan’s national consumption of whole grains.

Brown rice is among the most familiar whole grains in Japan, given that rice serves as the primary staple in the Japanese diet. Renowned for its abundant health benefits, brown rice has been linked to a decreased risk of type 2 diabetes in prospective cohort studies conducted among nurses and health professionals in the United States [5,11,12]. Moreover, a prior study using data from the National Health and Nutrition Examination Survey conducted in the United States in the late 2000s reported that only 3% of adults consumed brown rice [13,14]. Nevertheless, similar to the situation with whole grains overall, there is currently limited knowledge regarding the national-level consumption of brown rice in Japan, primarily because of a lack of data. Previous research on this topic in Japan is confined to a large-scale prospective cohort study, which demonstrated that white rice intake was associated with an increased risk of type 2 diabetes in women [15]. Additionally, a cohort study of factory workers suggested that consumption of brown rice or multigrain rice contributed to weight control [16].

In the current study, we conducted an analysis of brown rice consumption among adults in Japan, using food intake records from the National Health and Nutrition Surveys (NHNS). This study had three primary objectives. First, we aimed to examine national trends in brown rice consumption. Second, we sought to evaluate differences in food and nutrient intakes between consumers of brown and white rice. Third, we aimed to investigate the sociodemographic characteristics and health behaviors of brown rice consumers.

## 2. Materials and Methods

### 2.1. Data Source

We used individual-level data from the NHNS conducted between 2012 and 2019 [17]. The NHNS is an ongoing cross-sectional household interview and examination survey implemented annually by the Japan Ministry of Health, Labor, and Welfare. We limited the study period to 2012–2019 because individual-level data on dietary intake by food items were electronically available only for this period at the time of our study. We obtained official approval to access individual-level data in accordance with the Statistics Act [18]. No ethical review was required for this study because the use of NHNS data is exempt under the Ethical Guidelines for Medical and Biological Research Involving Human Subjects [19].

The methodological details of the NHNS have been documented [17,20]. The sampling frame comprised a list of all residential census enumeration areas, stratified across Japan’s 47 prefectures. Each census enumeration area included approximately 50 households. The surveys employed a stratified two-stage cluster sampling design to obtain nationally representative data from the non-institutionalized Japanese population. In the first sampling stage, census enumeration areas were randomly selected from each prefecture, and this sample was utilized for the Comprehensive Survey of Living Conditions conducted every June. Subsequently, in the second sampling stage, the census enumeration areas sampled for the Comprehensive Survey of Living Conditions were subdivided into unit blocks, with each block comprising 20 to 30 households. Unit blocks were then randomly chosen from each prefecture. All individuals aged ≥1 year old residing in a household within 300 sampled unit blocks were eligible for inclusion in the NHNS. The large-scale surveys conducted in 2012 and 2016 employed a stratified single-stage cluster sampling design to obtain expanded samples representative at the prefecture level; census enumeration areas were randomly drawn from those in each prefecture, and all individuals aged ≥1 year old residing in 475 selected census enumeration areas were eligible for participation in the surveys.

The NHNS comprised a dietary intake survey, a lifestyle survey, and a physical examination. For the dietary intake survey, a semi-weighed household dietary record was employed to assess dietary intake of individuals aged ≥1 year old on a single day in November, excluding Sundays and public holidays. Trained fieldworkers, such as registered dietitians, visited households to provide instructions on filling out a self-administered questionnaire. Household members responsible for meal preparation used a scale to weigh and record each food and beverage item consumed in the household, including food waste and leftovers. To estimate individual food intakes from shared dishes, approximate proportions of each food item were assigned across household members. For foods consumed away from home, household members reported portion sizes, amounts consumed, and leftovers. Fieldworkers revisited households to collect and review the questionnaires, clarify entries, fill in missing information, and correct errors. Subsequently, each food item was coded according to food codes of the Standard Tables of Food Composition in Japan 2010 edition (for the surveys from 2012 to 2017) and the 2015 edition (for surveys conducted in 2018 and 2019) [21,22].

### 2.2. Data Preparation

We restricted our study to individuals aged ≥20 years old. To estimate the distributions of brown rice and white rice intake, we obtained data from a sample of 84,377 participants aged ≥20 years old (45,519 females and 38,858 males) across surveys conducted from 2012 to 2019, after excluding 13,901 participants (14.1%) with missing values regarding food intake. The sample size for this study varied from 26,726 in 2012 to 4927 in 2019 (Table S1).

To investigate the characteristics of individuals who consume brown rice, we merged individual-level data from the NHNS conducted during 2013–2015 and 2017–2019 with socioeconomic information obtained from the household questionnaire of the Comprehensive Survey of Living Conditions. We excluded the large-scale NHNS in 2012 and 2016 from the record linkage process because their samples were not drawn from the master samples of the Comprehensive Survey of Living Conditions. Employing a two-stage record linkage method, we matched key variables including a prefecture identification number, de-identified codes on census enumeration districts, unit blocks, households and household members, sex, birth year and month, and age [23]. This yielded a merged dataset comprising 33,570 participants (17,939 females and 15,631 males) out of 35,800 participants aged ≥20 years with valid food intake records in 2013–2015 and 2017–2019. The merged sample size ranged from 4629 in 2019 to 6408 in 2014 (Table S1).

### 2.3. Measurement of Brown and White Rice Consumption

To quantify brown and white rice consumption, we utilized individual-level records of dietary intake by food items obtained from the survey. Participants were identified as consumers of brown rice and white rice if they had consumed at least one of the 15 non-glutinous rice items listed in Table S2. We excluded consumption of glutinous rice and other rice products to clarify trends and characteristics of brown rice consumption. Non-glutinous rice is more commonly consumed on a daily basis than glutinous rice in Japan. Glutinous rice is primarily reserved for making traditional Japanese sweets and for meals, especially during special occasions and festivals. Subsequently, we categorized participants into four groups on the basis of their rice intake: (1) consumers of only white rice, (2) consumers of only brown rice, (3) consumers of both types, and (4) non-consumers of either. Participants who exclusively consumed brown rice or combined it with white rice were classified as brown rice consumers. Weight change factors from Table S2 were applied to convert the weights of cooked rice items to their respective dry equivalents.

### 2.4. Statistical Analysis

We examined the percentiles of daily intake of brown and white rice for each survey year. Additionally, we calculated the percentages of the four consumption groups for brown and white rice, adjusting standard errors to account for the multi-stage sampling method involving stratification and clustering. Specifically, using data from the expanded survey in 2016, we compared mean intakes of nutrients between individuals who consumed brown rice and those who exclusively consumed white rice. We also examined differences in mean intakes of food items between the groups over time, including legumes, nuts, vegetables, fruits, fish, red meat, processed meat, and dairy products.

We performed a pooled analysis of data from surveys conducted during 2013–2015 and 2017–2019 to estimate the odds ratios for brown rice consumption using multivariable logistic regression (*n* = 31,675). Participants who abstained from both brown and white rice consumption were excluded from the regression analysis. The dependent variable was binary, indicating whether an adult consumed brown rice (coded as 1) or only white rice (coded as 0). Independent variables included sociodemographic characteristics (sex, age, municipality of residence, households without children aged under 6 years old, and educational background), health behaviors (body mass index, regular exercise, smoking status, and alcohol consumption), and survey year. Detailed information on the data sources and categories for these independent variables is provided in Table 1. To account for correlations within households, we adjusted for standard errors of odds ratios accordingly. All analyses were performed using Stata/MP 18 (StataCorp LLC, College Station, TX, USA). Statistical significance was defined as two-sided *p*-values < 0.05.

## 3. Results

The 95th percentile of daily brown rice intake among adults aged ≥20 years old consistently remained at 0.0 g throughout the study period (Table 2). The median daily intake of white rice decreased from 151.0 g (interquartile range: 95.2–214.3) in 2012 to 123.8 g (interquartile range: 71.4–190.5) in 2019. These trends were observed in both females and males.

Over 90% of adults consumed only white rice, with the percentage significantly declining from 94.0% (95% confidence interval [CI]: 93.6–94.4) in 2012 to 91.1% (95% CI: 89.9–92.2) in 2019 (Table 3). The percentage of brown rice consumers increased from 1.8% (95% CI: 1.6–2.1) in 2012 to 2.6% (95% CI: 2.0–3.4) in 2019, and the majority of this group consumed brown rice in combination with white rice. The percentage of adults who consumed neither type of rice significantly increased from 4.2% (95% CI: 3.9–4.5) in 2012 to 6.3% (95% CI: 5.5–7.2) in 2019. All of these trends were observed in both females and males. The percentage of brown rice consumers was significantly higher among female than male respondents, except in 2013 (*p* = 0.09), 2015 (*p* = 0.38), and 2018 (*p* = 0.06).

In 2016, both female and male consumers of brown rice had significantly higher mean intake levels of macronutrients, compared with white rice consumers, such as plant-based protein, plant-based fat, and omega-6 fatty acids, as well as dietary fibers, and most vitamins and minerals (Table 4). Notably, the mean intake of vitamins A, K, B12, and C, which are scarce in grains, was higher for brown rice consumers compared with those who consumed only white rice. Nevertheless, no significant differences were observed in mean intake levels of animal-based protein and fat, omega-3 fatty acids, cholesterol, carbohydrates, and sodium for both sexes between brown rice consumers and white rice consumers. Additionally, total energy, water, and vitamin D intake levels did not show significant differences based on rice consumption among male respondents.

**Figure 1:**
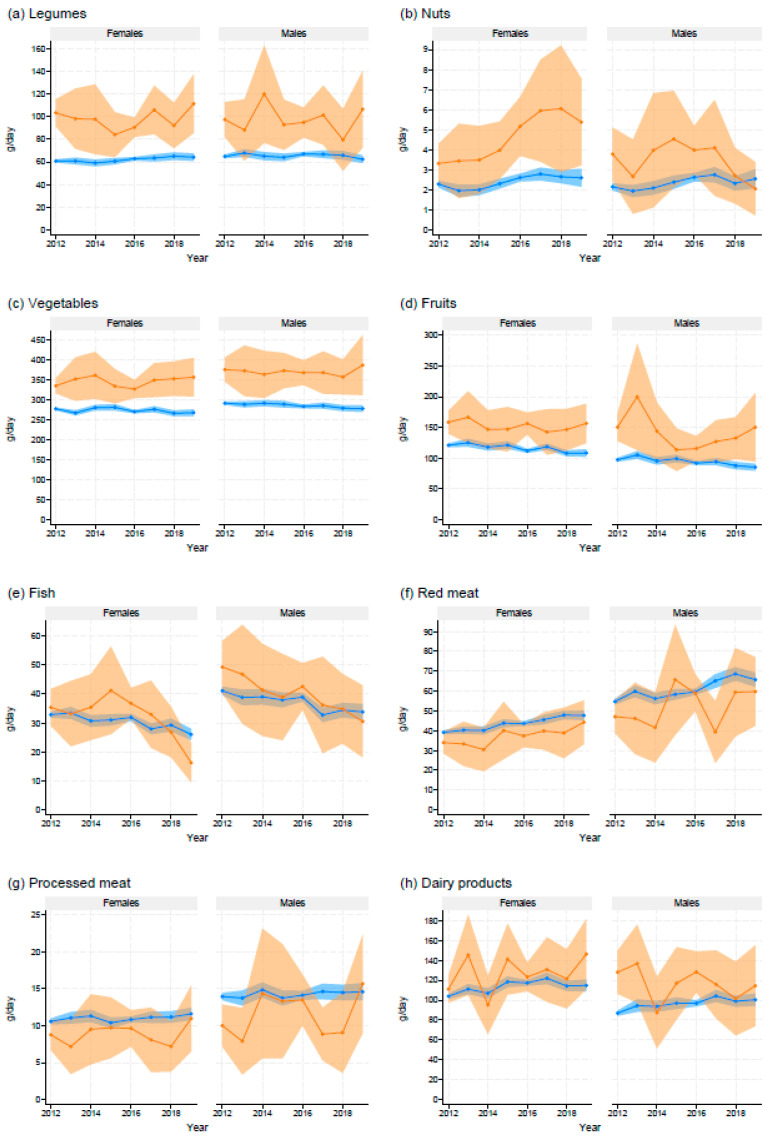
Mean daily food intake among adults aged ≥20 years consuming brown rice and those consuming only white rice, based on the National Health and Nutrition Surveys, Japan, 2012–2019. Orange color, brown rice consumers; blue color, white rice consumers. Shaded areas show the 95% confidence interval.

Brown rice consumers exhibited a significantly higher mean intake of legumes, vegetables, and fruits in both sexes, as well as nuts in female respondents, compared with those who consumed only white rice across all or most of the survey years (Figure 1). However, there was no significant difference in mean fish intake between the groups. Mean intakes of red meat and processed meat tended to be lower in brown rice consumers compared with white rice consumers, although statistical significance was not observed in most of the study period. Similarly, the mean intake of dairy products tended to be higher for brown rice consumers than white rice consumers, but this difference was not statistically significant in most of the study period.

After adjusting for confounding factors, brown rice consumption exhibited significant positive associations with certain sociodemographic characteristics (Table 5). These characteristics included female adults (*p* = 0.043), older adults (*p* = 0.003 for the 50–59 years and 60–69 years age groups, and *p* = 0.006 for the 70–79 years age group), residents of one of 21 major cities (*p* = 0.011), individuals living in households without children aged under 6 years old (*p* = 0.005), and those educated at a junior/career college or a university/graduate school (*p* < 0.001). Additionally, in terms of health behaviors, individuals were significantly more likely to consume brown rice if they had a lower body mass index (*p* = 0.002 for <18.5 kg/m^2^, and *p* < 0.001 for 18.5 to <25.0 kg/m^2^), engaged in regular exercise (*p* < 0.001), and were former or never smokers (*p* < 0.001).

## 4. Discussion

To the best of our knowledge, this study represents the first examination of the national trends and characteristics of brown rice consumption in Japan. Our results revealed that only 2% of the adult population incorporated brown rice either exclusively or alongside white rice in their diet, while approximately 90% consumed exclusively white rice. This level of brown rice consumption was close to national estimates in the United States during the 2000s, which ranged from 1% to 3% [13,14]. However, considering that rice serves as the main staple food in Japan, the adoption of brown rice consumption appears to lag even further behind that in the United States, in which rice is not a primary dietary staple for the majority of the population. The slow progress in embracing one of the most familiar whole grains may underscore the overall limited intake of whole grains in Japan. This evidence will be instrumental in formulating nutrition policies focused on whole grain intake in Japan, where there is currently no specific policy endorsing whole grains, despite the presence of official dietary recommendations in the United States and several other countries [26,27].

The current results support the potential advantages of incorporating brown rice into dietary patterns, because it correlates with increased consumption of plant-based foods such as vegetables, legumes, nuts, and fruits, all of which are essential components of a healthy, environmentally sustainable diet [2]. Notably, individuals who consume brown rice demonstrated a higher intake of vegetables and legumes compared with white rice consumers, highlighting their preference for plant-based nutrition. However, our analysis suggested that brown rice consumption was not consistently correlated with the intake of animal-source foods, including fish, red meat, processed meats, and dairy products. Specifically, brown rice consumers may not necessarily favor fish over red meat as a healthier protein source, which may appear counterintuitive considering the recommended role of fish in promoting cardiovascular health [28]. One plausible explanation is that both brown rice and white rice consumers consume fish to a similar extent, assuming the common dietary practice of pairing rice with fish [29]. This observation may align with broader national trends of declining fish consumption and rising meat consumption per capita in recent decades [30].

Our results revealed correlations between brown rice consumption and increased intake of essential nutrients, some of which are not naturally present in brown rice. Integrating brown rice into one’s diet may promote balanced nutrition by fostering the consumption of other plant-based foods, as mentioned earlier. A prior study conducted in the United States revealed that individuals who consumed either white or brown rice had greater intakes of macronutrients compared with non-rice consumers [13]. The current findings contribute to the existing literature by confirming that consumers of brown rice exhibit even higher intakes of essential nutrients than those who consume only white rice. These results may be helpful for clarifying the mechanism underlying the association between brown rice intake and a reduced risk of type 2 diabetes [11]. However, our analysis did not find any correlation between brown rice consumption and salt intake. A previous study based on the NHNS suggested that high intakes of rice, fish, and salt-based seasonings were part of a dietary pattern characterized by plant foods and fish [31]. From a practical public health perspective, advocating for salt reduction in conjunction with brown rice consumption would be an effective strategy for achieving Sustainable Healthy Diets and Planetary Health Diets.

Incorporating brown rice into the diet presents a challenge for many Japanese people. For example, cooking brown rice requires more time and attention compared with white rice. Additionally, the texture and flavor of brown rice may not be as universally appealing as those of white rice, posing difficulties for individuals with large families. Previous studies conducted in Asia, Latin America, and Africa have identified various barriers to the acceptance of brown rice, including limited awareness of its nutritional benefits, and its particular sensory attributes [32,33,34,35,36,37,38,39]. Practical constraints related to market availability, price, and cooking requirements also contribute to these barriers. These previous studies suggested various strategies to improve the acceptance of brown rice, such as health education to correct misperceptions regarding the quality of brown rice, introducing it at an early age through school lunches, and efforts to lower costs and increase availability.

A prior online survey conducted in the Greater Tokyo Area in 2018 revealed that 7% of respondents consumed brown rice three times per week or more frequently [40]. It highlighted a widespread recognition of the health benefits of brown rice, including its positive impact on health, rich nutrient content, and potential to improve bowel movements. However, some negative perceptions, such as bland taste and cooking difficulties, and unaffordability were also noted. Moreover, many respondents were unaware of technological advancements such as new varieties with improved digestibility and texture, as well as rice cookers equipped with brown rice mode. To increase awareness and affordability, enhanced efforts are necessary across various sectors, including the rice industry. Engaging influential figures such as researchers and culinary experts to disseminate information through active media reporting would further stimulate interest in the market. Promoting the consumption of processed brown rice and pre-germinated rice would help lessen perceived drawbacks of traditional brown rice, such as its challenges in preparation and consumption [41]. Providing brown rice through dining out, school meals, and prepared foods would encourage the younger generation to adopt a lifelong brown rice diet.

Our observations regarding the specific characteristics of brown rice consumers highlight key target groups for effectively increasing the prevalence of brown rice consumption. These groups include females, older individuals, residents of major cities, individuals with higher education levels, and those who lead healthier lifestyles. Disparities may exist across groups in terms of awareness of healthy diets and access to brown rice products. For example, compared with rural residents, residents of major cities may have greater access to nutritional information about brown rice and be able to purchase brown rice products that are readily available at local grocery retailers. Prioritizing the expansion of brown rice consumption within these target groups could facilitate the gradual dissemination of awareness regarding its benefits to other groups that are less informed or knowledgeable. Additionally, it should be noted that individuals living with very young children may tend to avoid brown rice because of concerns about its slower digestion and absorption, and the potential to cause stomach discomfort.

One of the strengths of the current study is the utilization of individual-level food intake records from national surveys, which allowed for the assessment of brown rice consumption across the entire country for the first time in Japan. Previous research on brown rice intake in the general population has been limited in Japan, with one cohort study focusing on employees of a food manufacturing company [16]. Additionally, national intake of brown rice has been examined in only a few studies in the United States using the National Health and Nutrition Examination Surveys [13,14]. Therefore, the current findings are novel, demonstrating the distribution of brown and white rice intake, as well as the sociodemographic characteristics associated with brown rice consumption in Japan.

The current study involved several limitations that should be considered when interpreting the results. First, the current findings were derived from cross-sectional data, limiting the ability to make causal inferences. The cross-sectional design precluded the establishment of any causal relationships between brown rice intake and individual characteristics or nutrient intake. Second, the response rates of households in the NHNS ranged from 44.4% in 2016 to 67.2% in 2014 [17]. These varying response rates, particularly on the lower end, may introduce selection bias and impact the generalizability of the findings. Third, in investigating the intake of brown rice, we did not consider the consumption of multigrain rice, although it also contains nutrients that may contribute to the prevention of noncommunicable diseases [16,42]. Future research should explore multigrain rice consumption, as it may offer additional insights into dietary patterns and health outcomes. Fourth, our analysis, based on individual-level data, did not examine macro-level cultural and traditional factors influencing brown rice consumption.

## 5. Conclusions

Although only a small minority of adults in Japan consume brown rice, it is associated with increased intake of plant-based foods and essential nutrients. The initial approach to promoting brown rice consumption should target individuals exhibiting sociodemographic characteristics and health behaviors that are conducive to incorporating it into their diets. Embracing a brown rice-based diet could ultimately contribute to achieving a more sustainable dietary pattern to promote planetary health and the environment. Given that white rice remains the primary choice for daily meals in Japan, future research should explore the impact of gradually replacing white rice with brown rice within the general population, particularly in terms of preventing type 2 diabetes and mitigating the rise of associated national healthcare expenditures.

## Figures and Tables

**Table 1:** Description of independent variables used in the multivariable logistic regression analysis of brown rice consumption, on the basis of data from the National Health and Nutrition Survey and the Comprehensive Survey on Living Conditions, Japan, 2013–2015 and 2017–2019.

Data Source, Characteristics, Values	Reference Categories
*National Health and Nutrition Survey*	
Sex	
Females, males	Males
Age, years	
20–29; 30–39; 40–49; 50–59; 60–69; 70–79; ≥80	20–29 years
Municipality of residence	
21 major cities; other cities; towns/villages	Other cities
Body mass index, kg/m^2 a^	
<18.5; 18.5 to <25.0; 25.0 to <30.0; ≥30.0; missing	≥ 25.0 kg/m^2^
Regular exercise habit ^b^	
Absent; present; missing	Absent
Smoking status ^c^	
Former/never smoker; daily/occasional smoker; missing	Daily/occasional smoker
Alcohol consumption (2014, 2015, 2017–2019) ^d^	
Non-drinker; drinker; missing	Drinker
*Comprehensive Survey on Living Conditions*	
Educational background	
Elementary/junior high school; senior high school; junior/career college; university/graduate school; unknown	Elementary/junior high school
Households without children aged <6 years	
Not applicable; applicable	Not applicable
Alcohol consumption (2013) ^d^	
Non-drinker; drinker; missing	Drinker
Survey year	
2013, 2014, 2015, 2017, 2018, 2019	2013

^a^ In the physical examination, standing height was measured to the nearest millimeter using a stadiometer while participants were barefoot. Weight was measured to the nearest 0.1 kg with participants wearing light clothing. Body mass index was calculated as weight in kilograms divided by the square of height in meters. ^b^ Participants in the physical examination were classified as regular exercisers if they reported consistently engaging in exercise sessions of at least 30 min, at least twice a week, for over a year [24]. ^c^ Participants were categorized as former/never smokers if they selected “Have smoked previously but not smoked for more than one month” or “Have never smoked,” and as daily/occasional smokers if they reported “Smoking daily” or “Smoking on occasion” in response to the question “Do you smoke?” in the lifestyle habits questionnaire. ^d^ Participants were classified as drinkers if they reported consuming alcoholic beverages equivalent to one or more cups of Japanese sake, typically containing 15% alcohol by volume and a volume of 180 mL each, for three days per week or more frequently [25]. The National Health and Nutrition Survey in 2013 did not include questions on alcohol consumption. Instead, data on alcohol drinking habits were obtained from the health questionnaire of the Comprehensive Survey on Living Conditions.

**Table 2:** Percentiles of daily intake of brown rice and white rice in grams among participants aged ≥20 years from the National Health and Nutrition Surveys, Japan, 2012–2019.

Year	n	Brown Rice Intake, Grams/Day									White Rice Intake, Grams/Day								
		Percentiles									Percentiles								
		1st	5th	10th	25th	50th	75th	90th	95th	99th	1st	5th	10th	25th	50th	75th	90th	95th	99th
Both sexes																			
2012	26,726	0.0	0.0	0.0	0.0	0.0	0.0	0.0	0.0	57.1	0.0	11.9	50.0	95.2	150.0	214.3	285.7	314.3	415.5
2013	6481	0.0	0.0	0.0	0.0	0.0	0.0	0.0	0.0	57.1	0.0	0.0	47.6	86.0	142.9	197.1	266.7	302.4	400.0
2014	6727	0.0	0.0	0.0	0.0	0.0	0.0	0.0	0.0	42.4	0.0	0.0	47.6	94.0	142.9	200.0	266.2	309.5	419.0
2015	6172	0.0	0.0	0.0	0.0	0.0	0.0	0.0	0.0	71.4	0.0	0.0	43.2	81.0	138.1	190.5	261.9	296.2	395.2
2016	21,851	0.0	0.0	0.0	0.0	0.0	0.0	0.0	0.0	66.7	0.0	0.0	47.6	85.7	142.9	192.4	261.9	295.2	393.3
2017	5750	0.0	0.0	0.0	0.0	0.0	0.0	0.0	0.0	66.7	0.0	0.0	41.0	76.2	132.9	190.5	257.1	291.4	392.9
2018	5743	0.0	0.0	0.0	0.0	0.0	0.0	0.0	0.0	71.4	0.0	0.0	41.9	80.1	132.4	190.5	259.5	295.2	395.2
2019	4927	0.0	0.0	0.0	0.0	0.0	0.0	0.0	0.0	66.7	0.0	0.0	38.1	71.4	123.8	190.5	246.7	285.7	372.9
Females																			
2012	14,461	0.0	0.0	0.0	0.0	0.0	0.0	0.0	0.0	60.0	0.0	0.0	47.6	76.2	128.6	176.2	225.7	259.5	309.5
2013	3483	0.0	0.0	0.0	0.0	0.0	0.0	0.0	0.0	66.7	0.0	0.0	40.5	71.4	114.3	166.7	214.3	247.1	290.5
2014	3615	0.0	0.0	0.0	0.0	0.0	0.0	0.0	0.0	47.6	0.0	0.0	42.9	71.4	117.1	166.7	209.5	238.1	285.7
2015	3332	0.0	0.0	0.0	0.0	0.0	0.0	0.0	0.0	71.4	0.0	0.0	33.3	71.4	109.5	166.7	214.3	242.9	309.5
2016	11,864	0.0	0.0	0.0	0.0	0.0	0.0	0.0	0.0	65.7	0.0	0.0	38.1	71.4	114.3	165.2	213.3	238.1	285.7
2017	3054	0.0	0.0	0.0	0.0	0.0	0.0	0.0	0.0	71.4	0.0	0.0	28.6	63.8	103.8	157.6	204.8	233.7	285.7
2018	3080	0.0	0.0	0.0	0.0	0.0	0.0	0.0	0.0	71.4	0.0	0.0	33.3	71.4	104.8	155.2	206.4	238.1	285.7
2019	2630	0.0	0.0	0.0	0.0	0.0	0.0	0.0	0.0	71.4	0.0	0.0	23.8	60.0	96.6	152.4	200.0	228.6	285.7
Males																			
2012	12,265	0.0	0.0	0.0	0.0	0.0	0.0	0.0	0.0	47.6	0.0	47.6	71.4	123.8	190.5	252.4	314.3	357.1	457.1
2013	2998	0.0	0.0	0.0	0.0	0.0	0.0	0.0	0.0	47.6	0.0	33.3	71.4	104.8	176.2	238.1	302.4	342.9	428.6
2014	3112	0.0	0.0	0.0	0.0	0.0	0.0	0.0	0.0	14.8	0.0	42.9	71.4	114.3	185.7	238.1	309.5	357.1	457.1
2015	2840	0.0	0.0	0.0	0.0	0.0	0.0	0.0	0.0	57.1	0.0	0.0	57.1	95.2	171.4	238.1	294.8	342.9	431.0
2016	9987	0.0	0.0	0.0	0.0	0.0	0.0	0.0	0.0	71.4	0.0	23.8	66.7	107.1	173.2	236.7	295.2	333.8	433.3
2017	2696	0.0	0.0	0.0	0.0	0.0	0.0	0.0	0.0	58.6	0.0	0.0	57.1	95.2	166.7	228.6	290.5	333.3	442.9
2018	2663	0.0	0.0	0.0	0.0	0.0	0.0	0.0	0.0	72.0	0.0	0.0	57.1	95.2	161.9	228.6	295.2	335.7	428.6
2019	2297	0.0	0.0	0.0	0.0	0.0	0.0	0.0	0.0	42.6	0.0	0.0	50.0	95.2	160.7	219.0	285.7	319.0	428.6

**Table 3:** Percentages of adults aged ≥20 years old by combination of brown rice and white rice consumption, estimated from the National Health and Nutrition Surveys, Japan, 2012–2019.

Year	Brown Rice			White Rice Only	Neither
	Total	Brown Rice only	Combined with White Rice		
Both sexes					
2012	1.8 (1.6, 2.0)	0.7 (0.6, 0.9)	1.1 (0.9, 1.3)	94.0 (93.6, 94.4)	4.2 (3.9, 4.5)
2013	1.6 (1.3, 2.1)	0.8 (0.6, 1.2)	0.8 (0.6, 1.2)	93.5 (92.7, 94.2)	4.9 (4.3, 5.6)
2014	1.6 (1.2, 2.0)	0.6 (0.4, 0.8)	1.0 (0.7, 1.4)	93.7 (92.8, 94.4)	4.8 (4.1, 5.5)
2015	2.4 (1.9, 3.1)	1.0 (0.7, 1.4)	1.5 (1.1, 2.0)	91.6 (90.5, 92.5)	6.0 (5.2, 6.9)
2016	2.3 (2.0, 2.6)	0.8 (0.7, 1.0)	1.4 (1.2, 1.7)	92.6 (92.1, 93.0)	5.2 (4.8, 5.6)
2017	2.2 (1.7, 2.8)	1.0 (0.7, 1.4)	1.2 (0.9, 1.6)	91.7 (90.8, 92.6)	6.1 (5.4, 6.9)
2018	2.6 (2.1, 3.2)	1.0 (0.8, 1.4)	1.5 (1.2, 2.1)	91.4 (90.4, 92.4)	6.0 (5.2, 6.9)
2019	2.6 (2.0, 3.4)	1.3 (0.9, 1.7)	1.4 (1.0, 1.9)	91.1 (89.9, 92.2)	6.3 (5.5, 7.2)
Females					
2012	2.0 (1.8, 2.3)	0.9 (0.7, 1.1)	1.2 (1.0, 1.4)	93.0 (92.5, 93.5)	4.9 (4.5, 5.4)
2013	1.8 (1.4, 2.4)	1.0 (0.7, 1.4)	0.8 (0.6, 1.2)	92.3 (91.3, 93.2)	5.9 (5.1, 6.8)
2014	1.9 (1.5, 2.5)	0.8 (0.5, 1.2)	1.1 (0.7, 1.7)	92.4 (91.2, 93.4)	5.7 (4.8, 6.7)
2015	2.6 (2.0, 3.2)	1.1 (0.7, 1.5)	1.5 (1.1, 2.0)	90.5 (89.3, 91.7)	6.9 (5.9, 8.0)
2016	2.4 (2.1, 2.8)	0.9 (0.8, 1.1)	1.5 (1.3, 1.8)	91.4 (90.8, 92.0)	6.1 (5.6, 6.7)
2017	2.5 (2.0, 3.2)	1.3 (0.9, 1.8)	1.2 (0.9, 1.8)	90.4 (89.2, 91.5)	7.1 (6.1, 8.1)
2018	2.9 (2.3, 3.6)	1.3 (0.9, 1.8)	1.6 (1.2, 2.2)	90.4 (89.1, 91.5)	6.8 (5.8, 7.9)
2019	3.2 (2.5, 4.1)	1.6 (1.1, 2.3)	1.6 (1.1, 2.2)	89.4 (87.9, 90.7)	7.4 (6.4, 8.6)
Males					
2012	1.5 (1.3, 1.8)	0.5 (0.4, 0.7)	1.0 (0.8, 1.2)	95.1 (94.7, 95.6)	3.3 (3.0, 3.7)
2013	1.4 (1.1, 1.9)	0.6 (0.4, 1.0)	0.8 (0.5, 1.2)	94.8 (93.9, 95.6)	3.7 (3.1, 4.5)
2014	1.2 (0.9, 1.7)	0.3 (0.2, 0.6)	0.9 (0.6, 1.3)	95.1 (94.2, 95.9)	3.7 (3.0, 4.5)
2015	2.3 (1.7, 3.1)	0.9 (0.6, 1.4)	1.4 (1.0, 2.0)	92.8 (91.6, 93.8)	4.9 (4.1, 5.9)
2016	2.1 (1.8, 2.4)	0.7 (0.6, 0.9)	1.4 (1.1, 1.6)	93.9 (93.3, 94.5)	4.0 (3.6, 4.5)
2017	1.8 (1.3, 2.4)	0.6 (0.4, 1.0)	1.1 (0.8, 1.7)	93.2 (92.1, 94.2)	5.0 (4.2, 5.9)
2018	2.3 (1.7, 3.0)	0.8 (0.5, 1.2)	1.5 (1.0, 2.2)	92.7 (91.5, 93.7)	5.1 (4.2, 6.1)
2019	2.0 (1.4, 2.7)	0.9 (0.6, 1.4)	1.1 (0.7, 1.7)	93.1 (91.8, 94.1)	5.0 (4.1, 5.9)

Values in parentheses indicate lower and upper ends of 95% confidence intervals.

**Table 4:** Mean daily nutrient intake among adults aged ≥20 years old consuming brown rice and those consuming white rice exclusively, on the basis of the National Health and Nutrition Survey, Japan, 2016.

	Females					Males				
	Brown Rice		White Rice Only		*p*-Value ^a^	Brown Rice		White Rice Only		*p*-Value ^a^
Total energy, kcal	1745.7	(26.9)	1692.5	(5.1)	0.049	2161.1	(40.8)	2117.4	(6.9)	0.288
Water, g	1604.6	(33.6)	1479.7	(7.5)	<0.001	1796.3	(47.9)	1723.6	(8.9)	0.139
Total protein, g	69.0	(1.2)	63.9	(0.3)	<0.001	82.8	(1.9)	76.2	(0.3)	<0.001
Animal-based protein, g	35.4	(1.0)	34.1	(0.2)	0.201	43.8	(1.5)	41.5	(0.3)	0.135
Plant-based protein, g	33.6	(0.6)	29.7	(0.1)	<0.001	39.1	(0.9)	34.8	(0.1)	<0.001
Total fat, g	54.8	(1.4)	51.7	(0.3)	0.029	66.4	(1.9)	60.6	(0.3)	0.002
Animal-based fat, g	24.9	(0.9)	25.7	(0.2)	0.344	33.1	(1.5)	31.1	(0.2)	0.181
Plant-based fat, g	30.0	(1.0)	26.0	(0.2)	<0.001	33.2	(1.2)	29.4	(0.2)	0.001
Saturated fatty acids, g	14.2	(0.4)	14.0	(0.1)	0.552	17.2	(0.6)	15.9	(0.1)	0.024
Monounsaturated fatty acids, g	18.5	(0.6)	17.5	(0.1)	0.101	23.1	(0.7)	21.1	(0.1)	0.007
Polyunsaturated fatty acids, g	12.5	(0.3)	11.1	(0.1)	<0.001	14.8	(0.5)	13.2	(0.1)	0.001
Omega-3 fatty acids, g	2.2	(0.1)	2.1	(0.0)	0.302	2.6	(0.1)	2.5	(0.0)	0.525
Omega-6 fatty acids, g	10.1	(0.3)	8.8	(0.0)	<0.001	12.0	(0.4)	10.5	(0.1)	<0.001
Cholesterol, mg	305.5	(10.0)	290.1	(1.8)	0.127	376.0	(16.7)	344.2	(2.2)	0.061
Carbohydrates, g	238.9	(4.1)	234.3	(0.7)	0.269	285.4	(6.3)	286.7	(1.0)	0.848
Total dietary fiber, g	19.5	(0.5)	14.3	(0.1)	<0.001	21.3	(0.6)	15.0	(0.1)	<0.001
Soluble dietary fiber, g	4.5	(0.1)	3.3	(0.0)	<0.001	4.8	(0.2)	3.4	(0.0)	<0.001
Insoluble dietary fiber, g	14.3	(0.3)	10.5	(0.1)	<0.001	15.7	(0.5)	11.0	(0.1)	<0.001
Vitamins										
Vitamin A, mcg RAE	642.9	(33.0)	502.1	(8.3)	<0.001	710.7	(57.8)	538.8	(9.8)	0.004
Vitamin D, mcg	9.3	(0.6)	7.6	(0.1)	0.004	9.1	(0.7)	8.4	(0.1)	0.254
Vitamin E, mg	8.0	(0.2)	6.3	(0.0)	<0.001	8.7	(0.3)	6.8	(0.0)	<0.001
Vitamin K, mcg	303.1	(13.6)	228.6	(2.1)	<0.001	347.4	(19.0)	247.3	(2.5)	<0.001
Vitamin B1, mg	1.0	(0.0)	0.8	(0.0)	<0.001	1.3	(0.0)	0.9	(0.0)	<0.001
Vitamin B2, mg	1.2	(0.0)	1.1	(0.0)	<0.001	1.4	(0.0)	1.2	(0.0)	<0.001
Niacin equivalents, mg	18.6	(0.4)	13.5	(0.1)	<0.001	22.6	(0.7)	16.5	(0.1)	<0.001
Vitamin B6, mg	1.5	(0.0)	1.1	(0.0)	<0.001	1.8	(0.1)	1.2	(0.0)	<0.001
Vitamin B12, mcg	6.7	(0.4)	5.8	(0.1)	0.030	8.2	(0.6)	7.0	(0.1)	0.043
Folate, mcg	337.1	(8.2)	282.8	(1.9)	<0.001	365.9	(11.9)	300.3	(2.1)	<0.001
Pantothenic acid, mg	6.3	(0.1)	5.1	(0.0)	<0.001	7.4	(0.2)	5.8	(0.0)	<0.001
Vitamin C, mg	120.5	(4.8)	97.6	(1.0)	<0.001	116.5	(5.8)	93.4	(1.0)	<0.001
Minerals										
Sodium, mg	3652.3	(88.5)	3630.0	(17.0)	0.805	4279.0	(121.9)	4273.9	(21.3)	0.967
Potassium, mg	2704.3	(55.4)	2206.1	(11.0)	<0.001	2960.0	(81.8)	2366.5	(12.0)	<0.001
Calcium, mg	580.6	(15.2)	491.2	(2.9)	<0.001	621.5	(20.9)	502.5	(3.2)	<0.001
Magnesium, mg	332.6	(6.6)	231.3	(1.0)	<0.001	386.2	(10.2)	260.2	(1.2)	<0.001
Phosphorus, mg	1147.3	(20.4)	918.7	(3.8)	<0.001	1352.2	(29.9)	1054.3	(4.4)	<0.001
Iron, mg	9.2	(0.2)	7.3	(0.0)	<0.001	10.4	(0.3)	8.1	(0.0)	<0.001
Zinc, mg	8.1	(0.1)	7.3	(0.0)	<0.001	9.9	(0.2)	8.9	(0.0)	<0.001
Copper, mg	1.3	(0.0)	1.1	(0.0)	<0.001	1.4	(0.0)	1.3	(0.0)	<0.001

Values in parentheses represent standard errors. ^a^ *p*-values for the difference in means between groups.

**Table 5:** Odds ratios for brown rice consumption among adults aged ≥20 years old, on the basis of data from the National Health and Nutrition Surveys and the Comprehensive Survey on Living Conditions, Japan, 2013–2015 and 2017–2019.

Characteristics	n	Bown Rice Consumers, n (%)	Odds Ratio (95% Confidence Interval)
Total	31,675	721 (2.3)	
*Sociodemographic characteristics*			
Sex			
Females	16,754	437 (2.6)	1.17 (1.00, 1.35)
Males	14,921	284 (1.9)	Reference
Age			
20–29 years	2267	37 (1.6)	Reference
30–39 years	3497	64 (1.8)	1.45 (0.92, 2.28)
40–49 years	4969	95 (1.9)	1.32 (0.85, 2.04)
50–59 years	4881	127 (2.6)	1.76 (1.22, 2.56)
60–69 years	6949	185 (2.7)	1.87 (1.23, 2.85)
70–79 years	6103	164 (2.7)	1.85 (1.19, 2.87)
≥80 years	3009	49 (1.6)	1.19 (0.71, 1.98)
Municipality of residence			
12 major cities	6196	185 (3.0)	1.36 (1.07, 1.72)
Other cities	21,843	463 (2.1)	Reference
Towns/villages	3636	73 (2.0)	1.03 (0.74, 1.44)
Households without children aged <6 years			
Not applicable	3035	38 (1.3)	Reference
Applicable	28,640	683 (2.4)	1.91 (1.22, 2.99)
Educational background			
Elementary/junior high school	4629	74 (1.6)	Reference
Senior high school	12,853	249 (1.9)	1.19 (0.87, 1.63)
Junior/career college	5406	164 (3.0)	1.90 (1.34, 2.70)
University/graduate school	6488	199 (3.1)	2.13 (1.49, 3.04)
Unknown	2299	35 (1.5)	0.90 (0.55, 1.47)
*Health behaviors*			
Body mass index			
<18.5 kg/m^2^	2045	58 (2.8)	1.71 (1.21, 2.41)
18.5 to <25.0 kg/m^2^	17,487	451 (2.6)	1.52 (1.20, 1.92)
25.0 to <30.0 kg/m^2^	5556	92 (1.7)	Reference
≥30.0 kg/m^2^	1053	16 (1.5)	0.96 (0.55, 1.68)
Missing	5534	104 (1.9)	1.48 (1.02, 2.15)
Regular exercise habit			
Absent	13,241	289 (2.2)	Reference
Present	5434	191 (3.5)	1.46 (1.19, 1.79)
Missing	13,000	241 (1.9)	0.83 (0.65, 1.07)
Smoking status			
Former/never smoker	25,100	668 (2.7)	2.74 (1.96, 3.82)
Daily/occasional smoker	5472	44 (0.8)	Reference
Missing	382	9 (2.4)	4.56 (1.90, 10.92)
Alcohol consumption			
Non-drinker	24,984	598 (2.4)	1.14 (0.92, 1.42)
Drinker	6199	114 (1.8)	Reference
Missing	492	9 (1.8)	0.72 (0.31, 1.66)
Survey year			
2013	5818	103 (1.8)	Reference
2014	6098	102 (1.7)	0.92 (0.64, 1.33)
2015	5502	144 (2.6)	1.39 (0.98, 1.98)
2017	4966	113 (2.3)	1.23 (0.86, 1.77)
2018	4956	137 (2.8)	1.51 (1.07, 2.14)
2019	4335	122 (2.8)	1.57 (1.10, 2.23)

## Data Availability

The availability of data analyzed in this study is restricted under the Statistics Act.

## Supplementary Materials

The following are available online at https://www.mdpi.com/article/10.3390/nu16101473/s1, Table S1: Number of participants aged ≥20 years in the National Health and Nutrition Survey and those included in the study with records merged from the Comprehensive Survey of Living Conditions; Table S2: Food items and weight change factors. This table presents the food items included in the study with their corresponding weight change factors, obtained from the Standard Tables of Food Composition in Japan, 2015 edition [22].
