# Supplementary material for: Trends and Characteristics of Brown Rice Consumption among Adults in Japan: An Analysis of the National Health and Nutrition Surveys, 2012–2019
Source: Nutrients. 2024 May 13;16(10):1473. doi: 10.3390/nu16101473 (PMC11124117; doi:10.3390/nu16101473)
Supplement: Supplementary file 1 [file nutrients-16-01473-s001.zip › nutrients-3015698-supplementary.pdf]

Supplementary material

Trends and characteristics of brown rice consumption among adults in Japan: an analysis of the National Health and Nutrition Surveys, 2012–2019

Nayu Ikeda, Miwa Yamaguchi, Nobuo Nishi

Correspondence: Nayu Ikeda, PhD
ikedan@nibiohn.go.jp
Section of Population Health Metrics
International Center for Nutrition and Information
National Institute of Health and Nutrition
National Institutes of Biomedical Innovation, Health and Nutrition
Settsu, Osaka, Japan

Table S1. Number of participants aged ≥20 years in the National Health and Nutrition Survey and those included in the study with records merged from the Comprehensive Survey of Living Conditions

Sex, survey year	n	Included in the study, n (%)		Merged with CSLC, n (%)	
Sexes combined					
2012	30,639	26,726	(87.2)	NA	
2013	7,248	6,481	(89.4)	6,120	(84.4)
2014	7,738	6,727	(86.9)	6,408	(82.8)
2015	7,194	6,172	(85.8)	5,852	(81.3)
2016	26,225	21,851	(83.3)	NA	
2017	6,775	5,750	(84.9)	5,292	(78.1)
2018	6,642	5,743	(86.5)	5,269	(79.3)
2019	5,817	4,927	(84.7)	4,629	(79.6)
Total	98,278	84,377	(85.9)	33,570	(81.1)
Females					
2012	16,412	14,461	(88.1)	NA	
2013	3,867	3,483	(90.1)	3,276	(84.7)
2014	4,121	3,615	(87.7)	3,429	(83.2)
2015	3,861	3,332	(86.3)	3,148	(81.5)
2016	14,093	11,864	(84.2)	NA	
2017	3,543	3,054	(86.2)	2,795	(78.9)
2018	3,547	3,080	(86.8)	2,820	(79.5)
2019	3,092	2,630	(85.1)	2,471	(79.9)
Total	52,536	45,519	(86.6)	17,939	(81.4)
Males					
2012	14,227	12,265	(86.2)	NA	
2013	3,381	2,998	(88.7)	2,844	(84.1)
2014	3,617	3,112	(86.0)	2,979	(82.4)
2015	3,333	2,840	(85.2)	2,704	(81.1)
2016	12,132	9,987	(82.3)	NA	
2017	3,232	2,696	(83.4)	2,497	(77.3)
2018	3,095	2,663	(86.0)	2,449	(79.1)
2019	2,725	2,297	(84.3)	2,158	(79.2)
Total	45,742	38,858	(85.0)	15,631	(80.6)

CSLC, Comprehensive Survey of Living Conditions; NA, not applicable.

Table S2. Food items and weight change factors. This table presents the food items included in the study with their corresponding weight change factors, obtained from the Standard Tables of Food Composition in Japan, 2015 edition [1].

Food item	Weight change factor
Brown rice	
Rice, paddy rice, brown rice, raw	Not applicable
Rice, short grain, paddy rice, brown, "meshi" (cooked rice)	210
Rice, short grain, paddy rice, brown, "zengayu" (gruel)	500
Rice, short grain, paddy rice, brown, "gobugayu" (diluted gruel)	1,000
Rice, short grain, paddy rice, brown, "omoyu" (thin gruel)	1,700
Rice, short grain, paddy rice, germinated brown rice, raw	Not applicable
Rice, short grain, paddy rice, germinated brown rice, "meshi" (cooked rice)	210
White rice	
Rice, paddy rice, non-glutinous, well-milled, raw	Not applicable
Rice, short grain, paddy rice, non-glutinous rice, well-milled, "meshi" (cooked rice)	210
Rice, short grain, paddy rice, well-milled, "zengayu" (gruel)	500
Rice, short grain, paddy rice, well-milled, "gobugayu" (diluted gruel)	1,000
Rice, short grain, paddy rice, well-milled, "omoyu" (thin gruel)	1,700
Rice, non-glutinous rice products, quick-cooking rice, regular, raw	210
Rice, non-glutinous rice products, "Onigiri" (rice ball)*	210
Rice, non-glutinous rice products, "Yaki-onigiri" (baked rice ball)**	210

* Without laver and filling; containing 0.5 g of salt per 100 g.

** Containing 6.5 g of common soy sauce per 100 g.

References

1. Ministry of Education, Culture, Sports, Science, and Technology. Standard tables of food composition in Japan - 2015 - (Seventh revised edition). 2015. Available online: https://www.mext.go.jp/en/policy/science_technology/policy/title01/detail01/1374030.htm (accessed on March 12, 2024).
